# Electrophysiological Studies in Patients with Pulmonary Hypertension: A Retrospective Investigation

**DOI:** 10.1155/2014/617565

**Published:** 2014-05-26

**Authors:** Dirk Bandorski, Jörn Schmitt, Claudia Kurzlechner, Damir Erkapic, Christian W. Hamm, Werner Seeger, Ardeschir Ghofrani, Reinhard Höltgen, Henning Gall

**Affiliations:** ^1^The German Centre for Lung Research (DZL), University of Giessen and Marburg Lung Centre (UGMLC), Klinikstrasse 33, 35392 Giessen, Germany; ^2^Medical Clinic 1, University Hospital Giessen and Marburg GmbH, Klinikstrasse 33, 35392 Giessen, Germany; ^3^Department of Cardiology, Kerckhoff Heart and Thorax Centre, Benekestrasse 2-8, 61231 Bad Nauheim, Germany; ^4^Medical Clinic III, St. Agnes Hospital Bocholt, Barloer Weg 125, 46397 Bocholt, Germany

## Abstract

Few studies have investigated patients with pulmonary hypertension and arrhythmias. Data on electrophysiological studies in these patients are rare. In a retrospective dual-centre design, we analysed data from patients with indications for electrophysiological study. Fifty-five patients with pulmonary hypertension were included (Dana Point Classification: group 1: 14, group 2: 23, group 3: 4, group 4: 8, group 5: 2, and 4 patients with exercised-induced pulmonary hypertension). Clinical data, 6-minute walk distance, laboratory values, and echocardiography were collected/performed. Nonsustained ventricular tachycardia was the most frequent indication (*n* = 15) for an electrophysiological study, followed by atrial flutter (*n* = 14). In summary 36 ablations were performed and 25 of them were successful (atrial flutter 12 of 14 and atrioventricular nodal reentrant tachycardia 4 of 4). Fluoroscopy time was 16 ± 14.4 minutes. Electrophysiological studies in patients with pulmonary hypertension are feasible and safe. Ablation procedures are as effective in these patients as in non-PAH patients with atrial flutter and atrioventricular nodal reentrant tachycardia and should be performed likewise. The prognostic relevance of ventricular stimulations and inducible ventricular tachycardias in these patients is still unclear and requires further investigation.

## 1. Introduction

Pulmonary hypertension (PH) is defined as mean pulmonary arterial pressure ≥25 mm Hg at rest and a pulmonary capillary wedge pressure ≤15 mm Hg in patients with precapillary forms and ≥15 mm Hg in postcapillary PH [1].

Five different pathophysiological underlying mechanisms leading to PH are identified and accordingly the current classification distinguishes five groups of PH [2].

Supraventricular arrhythmias are frequent in these patients as well as cases of sudden deaths. The prognostic implications, especially of documented ventricular arrhythmias, are uncertain. Few studies have investigated patients with PH and arrhythmias [3–5]. Supraventricular tachycardias were the most common arrhythmias. Four studies report on ablation procedures of typical right atrial flutter [6–9]. Ablation of atrioventricular nodal reentrant tachycardia (AVNRT) was successfully performed in four patients (100%) in Ruiz-Cano's study [7].

In general data on electrophysiological studies (EPS) in patients with PH are rare and studies investigating results of EPS in patients with PH for arrhythmias other than atrial flutter and AVNRT are lacking.

Our study retrospectively analysed the indications and results of EPS in patients with PH at two experienced PAH centres.

## 2. Methods

We retrospectively investigated all consecutive patients with PAH that had undergone electrophysiological study in two centres (University of Giessen, Medical Clinic 1 and Medical Clinic 2 and Kerckhoff Klinik, Bad Nauheim, Department of Cardiology) in the last fourteen years (2000–2013). Medical records of all included patients were screened for relevant data, including the following: demographic data, aetiology of PH, comorbidities, indications for EPS, procedure data and results from EPS, and additional data from echocardiography, right heart catheterisation, and clinical presentation were collected. Data collected within the six months prior to EPS were considered.

### 2.1. Electrophysiological Studies

Depending on the expected/documented arrhythmia, the appropriate catheter setup was chosen. In patients with supraventricular tachycardias, we used a 4-catheter approach (high right atrium (HRA), coronary sinus (CS), His bundle electrogram (HBE), and RV-apex) with the aim of inducing and mapping the tachycardia. At least dual AV-Node physiology and two echo beats must be shown if no tachycardia was inducible but documentation showed the typical pattern. Ablation of AVNRT was performed by 4 mm nonirrigated (50 W/50°C). Success was defined as noninducibility and slow-pathway ablation. In patients with atrial flutter, we used a two-catheter approach with duodecapolar catheter (HRA->coronary sinus); ablation catheters were either 8 mm nonirrigated (50 W/50°C) or 4 mm irrigated tip (40 W/43°C). Success was defined as bidirectional block of the cavotricuspid annulus (>150 ms).

Nonsustained ventricular tachycardia during Holter-ECG monitoring was assessed, relevant and leading to EPS when causing clinical symptoms. Ventricular stimulation in patients with nonsustained ventricular tachycardia in Holter-ECG monitoring was performed from RV-apex and RV-outflow tracts, BD 500 and 400 ms, and coupling of three extrastimuli with coupling intervals of a minimum of 200 ms.

In supraventricular tachycardias other than typical atrial flutter or AVNRT, as well as in patients with monomorphic ventricular extrasystoly, a 3D mapping system (Ensite/NAVX, St. Jude Medical) was used to locate the origin (LAT and propagation map).

### 2.2. Echocardiography Studies

The following diameters and valves were measured.

Left atrial diameter (edge-to-edge method, parasternal long axis view), right atrial area (four-chamber view, measured at end-systole), left ventricle diameter (short axis, measured at end-diastole), right ventricle diameter (four-chamber view, measured at the end-diastole), left ventricular ejection fraction (LV-EF, biplane Simpson method, 2- and 4-chamber view), tricuspid annular plane systolic excursion (TAPSE), Tei-Index, systolic pulmonary artery pressure (sPAP), tissue Doppler of the free wall of the right ventricle (S′), stenosis, and insufficiency of valves [10–13].

### 2.3. Right Heart Catheterization

Right heart catheterization was performed via the right jugular vein. The hemodynamic measurements included right atrial pressure (RAP), mean pulmonary pressure (mPAP), pulmonary capillary wedge pressure (PCWP), pulmonary vascular resistance (PVR), and cardiac output (thermodilution).

### 2.4. Statistical Analysis

Patient data are presented as absolute numbers (mean or median) and standard deviations (SDs) or interquartile ranges (IQRs). Comparisons between groups were done using *t*-tests or chi-square tests as appropriate. *P* values of <0.05 were considered statistically significant. Statistical calculations were performed using SPSS, version 21 (IBM Inc., Armonk, NY, USA).

The association of arrhythmias with cardiac diameter and measurements of right heart catheterization was assessed with Pearson's correlation coefficient.

## 3. Results

Fifty-five patients were included, fourteen patients with pulmonary arterial hypertension (group 1), 23 patients with PH associated with left heart disease (group 2), 4 patients with PH associated with lung disease (group 3), 8 patients with chronic thromboembolic PH (CTEPH, group 4; six patients suffered from persistent CTEPH after pulmonary artery embolectomy; two patients declined pulmonary artery embolectomy), 2 patients with miscellaneous PH (group 5), and 4 patients with exercised-induced PH [14].

Mean age was 65 ± 13 years (range 30–94). Patients walked 334 ± 98.5 m in six minutes. Ten patients (18.2%) had anamnesis of coronary heart disease (CHD) with myocardial infarction in 5 patients (data available for 51 patients) and 37 (67.3%) patients with arterial hypertension. Patients with CHD (*n* = 10) were older (73 ± 13 versus 64 ± 13 years, *P* = 0.043) than patients without CHD (*n* = 41).

A cardiac pacemaker was implanted in 3 patients and an ICD was implanted in 2 patients (Table 1).

### 3.1. Electrophysiological Studies

The most common arrhythmia leading to EPS was atrial flutter in 14 of the 55 investigated patients. Mapping and ablation were successful in 12/14 patients with complete bidirectional isthmus block using irrigated RF-energy. Due to a giant right atrium (68 mm), a bidirectional isthmus block could not be achieved. In one patient ablation was complicated by a third degree heart block necessitating cardiac pacemaker implantation.

The lowest success of ablation occurred in patients with atrial tachycardia without feasibility of ablation because of multifocal tachycardia and in other cases placement of catheter was not successful because of right atrial enlargement.

Atypical “incisional” atrial flutter (incisional reentry tachycardia, Table 2) after cardiac surgery (CABG and aortic valve replacement; pulmonary valve repair due to stenosis) was successfully treated with ablation in two patients.

In patients presenting with suspected atrioventricular nodal reentrant tachycardia (AVNRT), the diagnosis was confirmed and slow pathway ablation was performed successfully in all patients.

In 15 patients a programmed ventricular stimulation protocol was performed because of nonsustained ventricular tachycardia (nsVT) during Holter-ECG monitoring. VT or VF could not be induced during EPS in any of these patients.

Indications for EPS in patients with cardiac pacemakers were nonsustained VT (*n* = 2) and atrial tachycardia (*n* = 1). In patients with an ICD indications for ablation were a slow VT (*n* = 1) and an HBE ablation in terms of rate control in one patient suffering from permanent rapidly conducted (>100/min) atrial fibrillation, despite medical therapy.

Patients with history of myocardial infarction suffered from the following arrhythmias: atrial flutter (*n* = 1), nsVT (*n* = 1), syncope (*n* = 1), slow VT (*n* = 1), and “incisional” reentry tachycardia (*n* = 1).

Fluoroscopy time was 16 ± 14.4 minutes for all patients, 19.8 ± 17.4 minutes for patients of group 1, 18.4 ± 14.9 minutes for patients of group 2, and 15.2 ± 13.2 minutes for patients of group 4. No differences showed statistical significance (group 1 versus group 2, *P* = 0.865; group 1 versus group 4, *P* = 0.716; group 2 versus 4 group, *P* = 0.787). In patients with atrial flutter, fluoroscopy time was 21.9 ± 14.6 minutes, 7.3 ± 10.6 minutes in patients with nsVT, 28.5 ± 15.9 minutes in patients with atrial tachycardia, and 7.3 ± 5.9 minutes in patients with AVNRT.

In total 36 ablations were performed and 25 of them were successful. In 4 patients with enlarged right atria (RA 63 mm/68 mm/56 mm/no data available) placement of catheter was not successful. The indications and results of EPS are shown in Table 2.

### 3.2. Arrhythmias Related to the Pathomechanisms in Patients with Pulmonary Hypertension and Coronary Heart Disease

The number of patients with atrial flutter was similar in groups 1 and 2 and higher in patients of group 4. More patients with PH related to left heart disease (group 2) suffered from ventricular arrhythmias such as nonsustained VTs and slow VTs detected by Holter-ECG monitoring. The vast majority of patients with atrial tachycardia suffered from PH of groups 1 (*n* = 4, 28%) and 2 (*n* = 4, 17%), and most of the patients with AVNRT suffered from PH of group 1. Incidence of coronary heart disease was different in patients of the three PH groups (group 1: *n* = 1, 7%; group 2: *n* = 6, 26%; and group 4: *n* = 3, 37.5%, Table 3).

In patients with pulmonary hypertension associated with lung disease (*n* = 4), miscellaneous PH (*n* = 2), exercised-induced PH (*n* = 4) atrial flutter (group 3: 2 patients, group 5: 1 patient), and nsVT (group 3: 1 patient, group 5: 1 patient, exercised-induced PH: 3 patients) were the most common arrhythmias leading to EPS. Other indications for EPS were syncope (group 3: 1 patient) and atrial tachycardia (exercised-induced PH: 1 patient). None of the patients of these groups had anamnesis of coronary heart disease. In one patient, data relating to coronary disease were not available.

### 3.3. Hemodynamic Parameters Evaluated by Echocardiography and Right Heart Catheterization

Cardiac function and hemodynamic parameters were evaluated by echocardiography and right heart catheterization. Echocardiographic data within the last six months before EPS were available in 54 patients and right heart catheterization was performed in 45 patients. Few patients had middle- or high-grade reduction of LV-EF.

Taking CHD into consideration, more patients without CHD (non-CHD) showed normal- and middle-grate reduction of LV-EF (*P* = 0.081; CHD: normal: 40%, low-grade reduction: 20%, middle-grade reduction: 30%, high-grade reduction: 10% versus non-CHD: normal: 66%, low-grade reduction: 22%, middle-grade reduction: 5%, and high-grade reduction: 7%).

We could not find any clinical relevant correlation between the described atrial/ventricular arrhythmias and cardiac diameters/hemodynamic parameters measured by echo or right heart catheterization (LA (0.246), LV (0.138), RA (0.209), RV (0.018), sPAP (0.007), mPAP (0.35), PVR (0.276), TAPSE (0.171), LV-EF (0.271), LVH (0.034), or PCWP (0.12), Table 4).

## 4. Discussion 

The present study is the first to analyse indications and results of EPS in patients with different kinds of pulmonary hypertension over a long period in centres with high expertise for pulmonary hypertension and diagnosis and therapy of cardiac arrhythmias. The study revealed that the incidence of EPS in these patients is low, approximately 2.2% (55 patients underwent EPS/2500 patients with pulmonary hypertension). The low number of EPS in these patients reflects the lower incidence of arrhythmias in our collective in contrast to other studies. In accordance to studies of Luesebrink, Bradfield, Ruiz-Cano, and Showkathali EPS were successful (85%) in patients with atrial flutter [6–9].

Impossibility of placement of catheter because of right atrial enlargement was also described by Bradfield et al. [7].

In our study atrial flutter was the second-most frequent arrhythmia in all patients and most frequent arrhythmia in patients without CHD. The results are in accordance with other studies, which describe a high incidence of atrial flutter in their cohorts [3–5, 7–9].

It is noteworthy that in nearly half of the patients who underwent EPS, the indication was nonsustained VT revealed by Holter-ECG monitoring. The comparably high number of nsVT in our study could be achieved because patients underwent Holter-ECG monitoring over a minimum period of 24 hours. It is remarkable that nsVTs were present in 11 patients who were not suffering from coronary heart disease. A third of the patients with coronary heart disease of groups 1 and 2 and no patient from group 4 had nsVTs. More patients with CHD showed (middle- and high-grade) reductions in LV-EF known as a risk factor for ventricular arrhythmias. These findings indicate that, in patients with PH, mechanisms other than ischemia/coronary heart disease seem to be responsible for ventricular tachycardias. Umar et al. revealed in their study on rats with PH induced by subcutaneous injection of monocrotaline early after depolarisations (EADs) from right ventricular epicardial surface triggering VT [15]. VT was not inducible in any of the patients during EPS. The prognostic relevance of nsVTs in patients with PH and their therapy remains unclear.

The majority of patients (57%) with atrial tachycardia showed multifocal complex substrates; ablation could only be performed successfully in two individuals. Studies in patients with pulmonary hypertension and atrial tachycardias are lacking.

The treatment of AVNRT was feasible; the results were identical (100% success) to Ruiz-Cano's study [8].

Our study revealed that the numbers of EPS in patients with PH have been increasing since 2000. Potential reasons include an improvement of ablation techniques and studies demonstrate that EPS and ablations are possible with high rates of success, especially in patients with atrial flutter and AVNRT [6–9].

Fluoroscopy time did not differ between groups 1, 2, and 4 but was longer in patients with atrial arrhythmias in contrast to nsVTs and AVNRTs. We found longer fluoroscopy time than in Luesebrink's study (16 ± 14.4 minutes for all patients; atrial flutter: 21.9 ± 14.6 minutes versus 14.5 ± 8.9 min) but shorter than in patients of Bradfield's study (44 ± 20 minutes). It can be assumed that enlargement of the right atrium/ventricle is the reason for the differences between the fluoroscopy times in the studies, whereas Bradfield and Luesebrink do not give information about the diameter of the right atrium/ventricle [6, 7].

In our study we did not reveal correlations between the types of arrhythmias, left and right cardiac diameters, LVH, LV-EF, TAPSE, sPAP, mPAP, PVR, and PCWP.

## 5. Conclusion

EPS in patients with pulmonary hypertension is feasible and safe. Ablation procedures are effective in patients with atrial flutter and AVNRT. Atrial tachycardias are often multifocal without possibility for ablation. The prognostic relevance of ventricular stimulations and inducible VTs in these patients is unclear. In patients with PH and atrial fibrillation, studies are lacking.

## Figures and Tables

**Table 1:** Baseline characteristics.

	All patients (*n* = 55)
	*N* (%)
Gender (f/m)	28/27 (51/49)
Comorbidities	
Coronary heart disease	10 (18.2%)
	Unknown: 4 (7.5%)
History of MI	5 (9.1%)
CABG	6 (10.9%)
Arterial hypertension	37 (67%)
Diabetes mellitus	13 (23.6)
Medication	
Vitamin K-Antagonist	40 (72%)
ASS	10 (18.2%)
Beta-blocker	21 (38.2%)
ACE inhibitor	24 (43.6%)
AT1 blockers	7 (12.7%)
Aldosterone antagonist	24 (43.6%)
Diuretics	44 (80%)
Amiodoaron	10 (18.2%)
Calcium channel blocker	3 (5.5%)
Digitoxin/Digoxin	3/2 (5.5/3.6%)
Cardiac devices	
Cardiac pacemaker	3 (5.5%)
Indication	Bradyarrhythmia absoluta (*n* = 1), sick sinus syndrome (*n* = 1), bradycardia/tachycardia syndrome (*n* = 1)
ICD	2 (3.6%)
Indication	Primary prevention

MI: myocardial infarction, CABG: coronary artery bypass graft, ACE: angiotensin converting enzyme, AT1: angiotensin 1, and ICD: implantable cardioverter defibrillator.

**Table 2:** Indications and results of EPS.

Arrhythmia/symptoms (number of patients)	EPS/ablation successful (number of patients)	EPS/ablation not successful (number of patients)	Reason/comment (number of patients)
Atrial flutter (*n* = 14)	12	2	Successful ablation in 12 patients. Placement of catheter not successful (*n* = 1), third degree heart block (*n* = 1).
Atrial tachycardia (*n* = 11)	2	9	Successful ablation in 2 patients. Multifocal tachycardia and ablation are not feasible (*n* = 4), placement of catheter is not successful (*n* = 3), no entrainment (*n* = 1), origin at atrial septum without is ablation procedure (*n* = 1).
Atrial fibrillation	1 (AV-node ablation)	—	Successful ablation in 1 patient.
Incisional reentry tachycardia (*n* = 2)	2	—	Successful ablation in 2 patients.
AVNRT (*n* = 4)	4	—	Successful ablation in 4 patients.
Nonsustained VT (*n* = 15)	15	—	No induction of VT.
Monomorphic premature ventricular beats (*n* = 3)	3	1	Successful ablation in 2 patients (right ventricle outflow tract). In 1 patient multiple foci (origin in left and right heart), ablation is not feasible.
Slow VT (*n* = 2)	2	—	Successful ablation in 2 patients.
Syncope (*n* = 3)	3	—	Diagnosis of bradyarrhythmia (*n* = 2), no reason detectable (*n* = 1).

EPS: electrophysiological study, VT: ventricular tachycardia, and AVNRT: atrioventricular nodal reentrant tachycardia.

**Table 3:** Arrhythmias related to the pathomechanisms in patients with pulmonary hypertension and coronary heart disease.

Arrhythmia (indication for EPS)	Group 1	Group 2	Group 4
Atrial flutter	3	4 (*n* = 1)	4 (*n* = 1)
Atrial tachycardia	4	5 (*n* = 1)	1
Atrial fibrillation	—	1	—
Incisional reentry tachycardia	—	2 (*n* = 1)	—
AVNRT	3	1	—
Nonsustained VT	3 (*n* = 1)	7 (*n* = 2)	—
Monomorphic premature ventricular beats	1	1	1 (*n* = 1)
Slow VT	—	2 (*n* = 1)	—
Syncope	—	—	2 (*n* = 1)

EPS: electrophysiological study, VT: ventricular tachycardia, AVNRT: atrioventricular nodal reentrant tachycardia, *n*: Number of patients with coronary heart disease with coronary heart disease.

**Table 4:** Hemodynamic parameters.

	All patients	Patients with PH group 1 (*n* = 14)	Patients with PH group 2 (*n* = 23)	Patients with PH group 4 (*n* = 8)
Characteristics				
Age	64	59	67	62
Female	28	8	11	3
CHD	10	1	6	3
Echocardiography				
sPAP (mm Hg)	58	66	52	68
LA-diameter (mm)	50	41	60	41
LV-diameter (mm)	49	44	53	45
LV-EF (reduction)	No: 32	No: 13	No: 7	No: 4
	Low: 13	Low: 1	Low: 9	Low: 2
	Middle: 5	Middle: —	Middle: 3	Middle: 2
	High: 4	High: —	High: 4	High: —
RA-diameter (mm)	58	61	57	59
RV-diameter (mm)	43	46	38	50
TAPSE (mm)	16.5	16	16	15
TEI-Index	0.55	0.6	0.52	0.55
AT (msec)	81	73	79	73
LVH (*n*)	17	1	10	3
Right heart catheterization				
PCWP (mm Hg)	15	9	22	10
mPAP (mm Hg)	42	43	41	42
PVR (dyn s cm^−5^)	647	835	515	640
RAP (mm Hg)	9	8	11	9
CO (L/min)	3.9	3.5	3.9	4.1

CHD: coronary heart disease, sPAP: systolic pulmonary arterial pressure, LA/RA=left/right atrium, LV/RV: left/right ventricle, EF: ejection fraction, TAPSE: tricuspid annular plane systolic excursion, AT: acceleration time, LVH: left ventricle hypertrophy, PCWP: pulmonary capillary wedge pressure, mPAP: mean pulmonary arterial pressure, PVR: pulmonary vascular resistance, RAP: right atrial pressure, and CO: cardiac output (litres (L) per minute (min)).
